# Predicting the Proliferation of Tongue Cancer With Artificial Intelligence in Contrast-Enhanced CT

**DOI:** 10.3389/fonc.2022.841262

**Published:** 2022-04-08

**Authors:** Ting-Guan Sun, Liang Mao, Zi-Kang Chai, Xue-Meng Shen, Zhi-Jun Sun

**Affiliations:** ^1^ The State Key Laboratory Breeding Base of Basic Science of Stomatology (Hubei-MOST) and Key Laboratory of Oral Biomedicine, Ministry of Education, School and Hospital of Stomatology, Wuhan University, Wuhan, China; ^2^ Department of Oral Maxillofacial-Head Neck Oncology, School and Hospital of Stomatology, Wuhan University, Wuhan, China

**Keywords:** tongue cancer, artificial intelligence, convolutional neural networks, proliferation, bioinformatics

## Abstract

Tongue squamous cell carcinoma (TSCC) is the most common oral malignancy. The proliferation status of tumor cells as indicated with the Ki-67 index has great impact on tumor microenvironment, therapeutic strategy making, and patients’ prognosis. However, the most commonly used method to obtain the proliferation status is through biopsy or surgical immunohistochemical staining. Noninvasive method before operation remains a challenge. Hence, in this study, we aimed to validate a novel method to predict the proliferation status of TSCC using contrast-enhanced CT (CECT) based on artificial intelligence (AI). CECT images of the lesion area from 179 TSCC patients were analyzed using a convolutional neural network (CNN). Patients were divided into a high proliferation status group and a low proliferation status group according to the Ki-67 index of patients with the median 20% as cutoff. The model was trained and then the test set was automatically classified. Results of the test set showed an accuracy of 65.38% and an AUC of 0.7172, suggesting that the majority of samples were classified correctly and the model was stable. Our study provided a possibility of predicting the proliferation status of TSCC using AI in CECT noninvasively before operation.

## Introduction

Tongue squamous cell carcinoma (TSCC) is the most common oral malignancy with increasing prevalence, and the 5-year survival rate is approximately 56.3% in China (1, 2). As a type of cancer, its fundamental trait is the ability to sustain proliferation (3), which relates closely to the tumor microenvironment. Tumors can produce mutations with neoantigens constantly during proliferation, influencing patients’ response to chemotherapy and immune checkpoint inhibitors (4). Therefore, with the advent of immunotherapy in cancer therapy, accurate prediction of the proliferation status is of great significance to the subsequent clinical strategy making.

Among the biomarkers correlated to the proliferation, the most frequently used one is the percentage of Ki-67 expression, quantified by the Ki-67 index. Ki-67 is a DNA-binding protein located in the nucleus, and is only expressed in the proliferation phase with a short half-life (5). High expression of the Ki-67 suggests active cell proliferation, and may lead to tumor invasion, lymph node metastasis, and poor prognosis (6–8). OSCC patients with higher Ki-67 expression had better response to radiosensitivity (9). In renal cancer, researchers found that Ki-67 could be a promising target for target therapy (10). In breast cancer, the Ki-67 index was considered as an indicator to monitor patient’s treatment effect to chemotherapy (11). However, the present procedure to acquire the Ki-67 index is biopsy-based immunohistochemical (IHC) staining (12). Not only is it invasive and time-consuming, its result is also greatly affected by the quality of the biopsy (13). Therefore, obtaining proliferation status by noninvasive methods before operation is important for the multi-disciplinary treatment of TSCC.

Compared to traditional biopsy-based IHC that only reflects parts of tumor, radiological images have the advantage of involving the entire tumor information in a non-invasive manner, avoiding the influence of tumor heterogeneity (14). Recently, artificial intelligence (AI) has shown outstanding performance in quantifying and analyzing patterns of tumor phenotypes on radiographic images with bioinformatics tools to build pathoclinical relevant models (15, 16). However, the capability of the AI model to noninvasively predict the proliferation status of TSCC using radiological images remains unclear. Therefore, in this study, our purpose is to develop and validate a classifier based on a relatively mature convolutional neural network (CNN) model: Inception-Resnet-V2 (17), and use it to predict the proliferation status of TSCC through contrast-enhanced CT (CECT). We adapted our CNN model to single CT slide of TSCC and hypothesized that by learning to detect subtle patterns, our model can build a bridge between CT images and proliferation status of TSCC.

## Materials and Methods

### Study Population

The data of the patients with TSCC in this experiment are collected from the Hospital of Stomatology, Wuhan University from August 2012 to July 2021. All the procedures were done following the Declaration of Helsinki guidelines and the protocol was ratified by the Institutional Medical Ethics Committee of School and Hospital of Stomatology, Wuhan University (2018LUNSHENZIA28).

Exclusion criteria were as follows:

- Patients without CECT images;
- Patients without Ki-67 staining;
- Artifacts caused by metal prostheses, implants, and titanium plate more than 50% in the lesion area;
- Patients with nonprimary tumor;
- Lesion is too small to be noticed in CECT.

**Figure 1:**
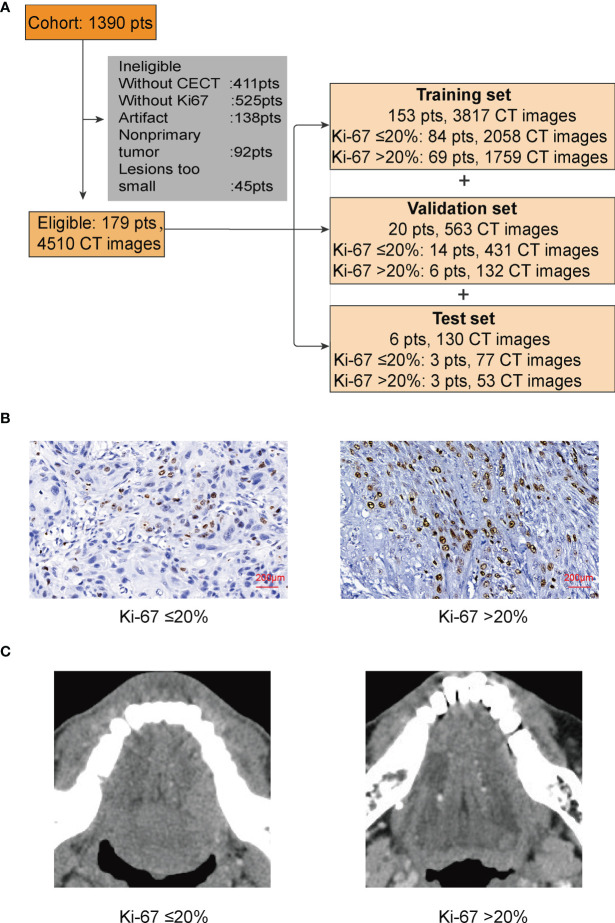
**(A)** In this study, patients (pts) were randomly divided into a training set, a validation set, and a test set. **(B)** IHC of Ki-67. We used 20% as the cutoff to divide our patients into two groups: low proliferation status with Ki-67 ≤ 20%; high proliferation status with Ki-67 >20%. **(C)** The lesion part of TSCC in CECT.

There were 1,390 patients diagnosed with TSCC in the Hospital of Stomatology, Wuhan University from August 2012 to July 2021. At present, we found 179 patients who met our requirements. All the included TSCC patients underwent primary surgery. The patients were randomly divided into a training set, a validation set, and a test set (**Figure 1A**).

### Immunohistochemistry of Ki-67 and Scoring

Immunohistochemistry (IHC) was established as previously described (18). Briefly, 4-μm-thick paraffin sections were deparaffinized in xylene and rehydrated *via* step-gradient ethanol. Antigen retrieval was operated with ethylenediaminetetraacetic acid, and the endogenous peroxidase was blocked by 3% hydrogen peroxide. After blocking with goat serum at 37°C for 1 h, sections were incubated with anti-human Ki-67 (clone: MXR002; RMA-0731, MXB Biotechnologies, China) with a dilution of 1:400 at a 4°C refrigerator overnight. Then, secondary biotin-labeled antibody and avidin-biotin-peroxidase reagent were used to incubate the sections at 37°C for 20 min each in order. Lastly, DAB kit (DAB-0031 MXB Biotechnologies, China) was applied for staining. The Ki-67 index was reported independently by the Department of Pathology in the Hospital of Stomatology, Wuhan University, without seeing patients’ CECT.

### CT Acquisition and Image Processing

All the CECT images in this study were obtained from a brightspeed 16-slice multi-slice CT machine (GE Healthcare, Milwaukee, WI) in the Hospital of Stomatology, Wuhan University (120 kV, 300 mA, slice thickness of 0.6 mm, pitch of 1.75:1, matrix of 512 × 512). The contrast agent is iopromide (Ultravist^®^300 Bayer AG, China), with a concentration of 300 mg/ml, 1.5–2.0 ml/kg.

To better process our data and reduce the computation cost, we converted the original CT files from DCM to PNG. The pixels of images were still 512 × 512 × 3, because besides the tongue, other unnecessary parts of the head in the CT images would disturb the judgment of CNN and bring unnecessary calculation. We then cut the majority of the noise parts out and only kept those images with lesion for the next step. After cutting, the size of the images was 180 × 180 × 3; only 12.36% of the original data remained (**Figure 1C**).

### Code Availability and CNN Training

The computer used in this study included an Intel i7 1070 4.20 GHz CPU with 16 GB DDR4 Memory and an NVIDIA GeForce GTX 1070 8 GB GPU. The programming language for neural network structure is tensorflow2.2 available on an open-source software pycharm 2021.1.1.

**Figure 2:**
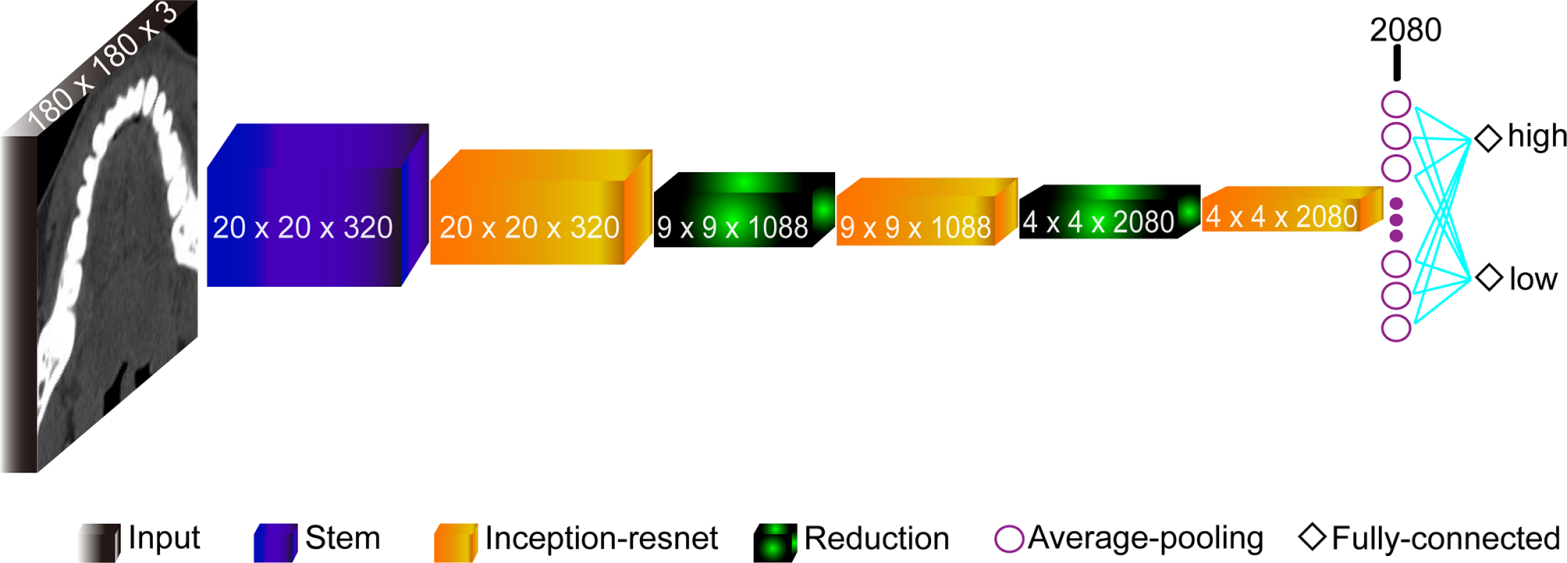
The structure of the Inception-Resnet-V2 network model.

The model based on the Inception-Resnet-V2 network was built with the structures shown in **Figure 2**. It consisted of 9 parts: the Input layer, the Stem layer, 3 Inception-resnet layers (Inception-resnet A, Inception-resnet B, and Inception-resnet C), 2 Reduction layers (Reduction A and Reduction B), the Average-pooling layer, and the Fully-connected layer (19). The input was the processed CT images with a size of 180 × 180 × 3. In addition, we also applied transfer learning to train our model on a large dataset on ImageNet in order to tackle the problem of limited dataset. With weights model adjusted properly, the pre-trained model can extract features and retrieve information from data automatically, and learn advanced abstract representations of data.

### Statistical Analysis and Evaluation Metrics


$$\text{Accuracy}\;=\;\frac{\text{TP}+\text{TN}}{\text{TP}+\text{TN}+\text{TP}+\text{FN}}$$

$$\text{Sensitivity}=\frac{\text{TP}}{\text{TP}+\text{FN}}$$

$$\text{Specificity}=\frac{\text{TN}}{\text{TN}+\text{FP}}$$

(TP: true positive, FP: false positive, TN: true negative, FN: false negative)

We used accuracy, loss, sensitivity, specificity, the receiver operating characteristic (ROC) curve, and AUC to assess the performance of our model. For statistical analysis, we regarded low proliferation as positive and high proliferation as negative. For the analysis of clinical and pathologic features, *χ* ^2^ test, Fisher’s exact test, and Mann–Whitney *U* test (*p* < 0.05) were performed using SPSS (IBM^®^ SPSS^®^ version 20 for Windows). This study was done following the TRIPOD reporting guidelines (**Supplementary Table 1**) (20).

## Results

### Patient Cohort and Ki-67 Immunostaining

From August 2012 to July 2021, a total of 1,390 patients were diagnosed with TSCC in the Hospital of Stomatology, Wuhan University. We collected data of 179 patients with TSCC meeting our criteria. We used pTNM classification in this study following the 8th edition of the AJCC staging standard (21). The median age was 53 years (range, 26–87), and gender ratio (male:female) was 119:60. There were 39 cases with excellent differentiation, 98 cases with moderate differentiation, and 42 cases with poor differentiation. There were 62 cases with tumor at the T1 stage, 81 cases with tumor at the T2 stage, and 36 cases with tumor at the T3 or T4 stage. Seventy-five patients were diagnosed with lymph node metastasis (**Table 1**).

**Table 1:** Clinicopathological parameters and proliferation status.

	Proliferation		*p*-value
	High	Low	
Sex			
Male	51	68	0.785^ns^
Female	27	33	
Age			
Median (range)	54 (26–87)	52 (28–74)	0.012*
Differentiation			
Well	10	29	<0.001***
Moderate	39	59	
Poor	29	13	
T stage			
T1	27	35	0.792^ns^
T2	37	44	
T3+T4	14	22	
Lymph node metastasis			
Negative	40	65	0.078^ns^
Positive	38	36	

*p < 0.05, ***p < 0.001, ns, not significant.

There is no explicit standard cutoff of Ki-67 index to divide TSCC into high and low proliferation status. In order to obtain a more balanced dataset, the median 20% (range, 0–80%) was used as the cutoff in this study (**Figure 1B**) (22). There were 56.4% of cases considered as low proliferation status (Ki-67 index ≤ 20%) and 43.6% of cases considered as high proliferation status. We connected proliferation status of TSCC with patients’ clinicopathological parameters, such as age, gender, T stage of tumor, lymph node metastasis, and tumor differentiation. Results in **Table 1** shows no difference in gender ratio, T stage of tumor, and lymph node metastasis with proliferation status. However, difference existed between proliferation status and age (*p* = 0.012), and between proliferation status and differentiation (*p* < 0.001).

### Predictive Power of the CNN Model

**Figure 3:**
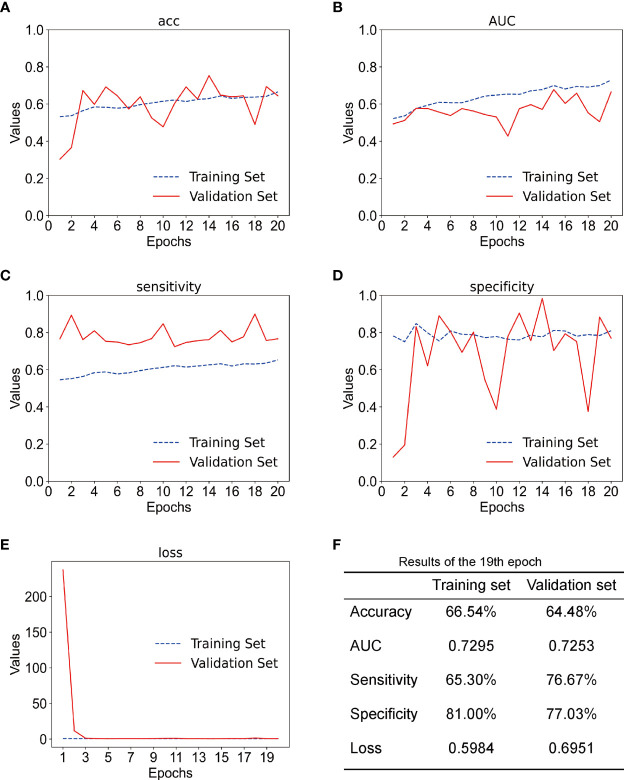
**(A)** The accuracy of training set and the validation set; acc stands for accuracy. **(B)** The AUC of the training set and the validation set. **(C)** The sensitivity of training set and validation set. **(D)** The specificity of the training set and the validation set. **(E)** The loss of the training set and the validation set. **(F)** Results of the training set and the validation set in the 19th epoch.

**Figure 4:**
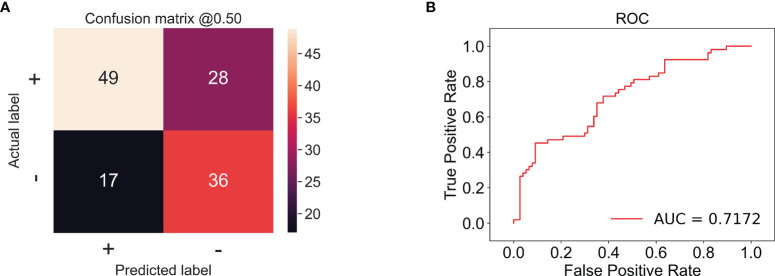
**(A)** The confusion matrix of the test set; the accuracy was 65.38%. + stands for low proliferation status. - stands for high proliferation status. The number in the matrix stands for CT amounts. **(B)** The receiver operating characteristic (ROC) example of the test set; the AUC is 0.7172.

In this experiment, we used CECT of patients with TSCC to train an Inception-ResNet-V2 network model. The training was stopped after 20 epochs because the results were flattening. The performance of our model in the training set and the validation set is shown in **Figure 3**. Our model performed best in the 19th epoch shown in **Figure 3F**. We then used the trained model to predict the test set with an accuracy of 65.38% (**Figure 4A**) and an AUC of 0.7172 (**Figure 4B**).

## Discussion

Uncontrolled proliferation is the most fundamental feature of cancers (3). It has a great impact on the immune microenvironment and immunotherapy by creating more mutations with neoantigens and damaging the function of T cells to help tumor escape immunity (4, 23, 24). A recent work also showed that PD-L1 expression was associated with a higher Ki-67 index in pituitary adenomas (25). On the other hand, patients with high proliferative oral cancers have higher recurrence risk after surgery, which is a potential indicator for extensive surgery and neck dissection (26, 27). Therefore, the proliferation status and Ki-67 index can help us develop therapeutic strategies, predict the therapy response, and observe the treatment effect.

In this study, we found that high proliferation status was associated with poor differentiation and old age of patients with TSCC, which are predictive factors of poor prognosis (28). However, the proliferation status showed no difference in T stage and lymph node metastasis in our study. On the contrary, Liu et al. came out with a different conclusion that the Ki-67 index had predictive value of lymph node metastasis in head and neck squamous cell carcinoma (29). The difference in results could be attributed to our different sample sizes, grouping cutoffs, and statistical methods. A recent study suggested that the expression of Ki-67 was strongly connected with tumor size and survival of patients (22).

The predictive results of our experiment proved that the CNN model was capable to predict the proliferation status of TSCC, with an accuracy of 65.38% and an AUC of 0.7172 in the test set, suggesting that the majority of our samples were classified correctly and the model was stable. Compared to radiomics, CNN is a more powerful model in processing data with grid patterns, especially images. Its spatial hierarchy enables it to automatically and adaptively learn features indistinguishable by human eyes from low-level patterns to high-level patterns. Accordingly, manual annotation is not considered indispensable, which makes prediction more efficient (30). Compared to other networks, Xie et al. found that the Inception-ResNet-V2 network could extract much more informative features from CT images of breast cancer (19). CECT can indicate the blood supply of tumor through the enhancement of blood vessels; therefore, it works well in reflecting soft tissue and providing inner information of tumor (31). Yoshiko Ariji et al. used CNN to assess the cervical lymph node metastasis of patients with oral cancer, acquiring an accuracy of 78.2% in their study (32). Indeed, MRI is superior to CT for imaging soft tissue. However, compared to MRI, CT has advantages of lower price, shorter inspection times, and less contraindications, especially for patients with dental artifacts (33). In some studies, CECT outperforms MRI in detecting neck metastasis and assessment of tumor depth of invasion in oral cancer (34, 35). Hence in TSCC, CECT is used more widely than MRI, which makes the research of CECT in TSCC more meaningful. In this study, all the CT images were obtained from the same CT machine, which could reduce bias of different machines.

Still, there remained several limitations in this study. First the rather small size of our study sample is a main issue. In the present study, only 179 patients and 4,510 CT images are involved. In a similar study of lung cancer, Fu et al. conducted radiomics to predict the Ki-67 expression of 282 patints with lung cancers, reaching an accuracy of 79.8% (36). We attributed the main reason to the size of our dataset, because when the sample size is small and asymmetrical, results of the AI model would come with bias, especially in the medical field (37, 38). Second, in the present study, only one model was used for analysis. The capability of other models needs further exploration. Third, we only used CECT in this study; patient’s clinical information was not involved. Patient’s clinical information such as age is correlated to the proliferation of TSCC, which we believe could assist the model to predict. Therefore, we considered this study as a preliminary exploratory study on the relationship between radiological images and the proliferation of TSCC. Further studies with an increased sample size and that involve more clinical information to improve the performance and universality of the AI model need to be conducted.

In conclusion, the present study provides a new way to establish the relationship between medical images and the pathological proliferation status of TSCC. To advance ideas on noninvasive and multidisciplinary integrative medicine, more researches are needed to reflect on the pathological features of cancer using AI.

## Data Availability Statement

The datasets presented in this article are not readily available because we want to protect the interest of the patients. Requests to access the datasets should be directed to Z-JS, sunzj@whu.edu.cn.

## Ethics Statement

The studies involving human participants were reviewed and approved by the Institutional Medical Ethics Committee of School and Hospital of Stomatology, Wuhan University (2018LUNSHENZIA28). The patients/participants provided their written informed consent to participate in this study.

## Author Contributions

T-GS and LM, contributed to conception, design, data acquisition, analysis, and interpretation, and drafted and critically revised the manuscript. Z-KC and X-MS contributed to data acquisition and data interpretation, and drafted and critically revised the manuscript. Z-JS contributed to conception, design, data analysis and interpretation, and drafted and critically revised the manuscript. All authors contributed to the article and approved the submitted version.

## Funding

This work was supported by the National Natural Science Foundation of China 82072996 and 82103333, and the Fundamental Research Funds for the Central Universities (2042021kf0174 and 2042021kf0216).

## Conflict of Interest

The authors declare that the research was conducted in the absence of any commercial or financial relationships that could be construed as a potential conflict of interest.

## Publisher’s Note

All claims expressed in this article are solely those of the authors and do not necessarily represent those of their affiliated organizations, or those of the publisher, the editors and the reviewers. Any product that may be evaluated in this article, or claim that may be made by its manufacturer, is not guaranteed or endorsed by the publisher.
